# The Accessory Sinuses of the Nose

**Published:** 1902-06

**Authors:** 


**The Accessory Sinuses of the Nose.** By A. LOGAN TURNER, M.D. Pp. xiv., 211. Edinburgh: William Green & Sons. 1901.

In the earlier chapters the author discusses somewhat fully and with great clearness the anatomy of the various accessory nasal cavities. Then follows an extremely interesting chapter on the comparative anatomy of the frontal sinuses in human crania. This section of the work is practically the subject matter of a lecture formerly delivered by Dr. Turner to the Royal College of Surgeons, and his later prize essay on "The Racial Characteristics of the Frontal Sinuses." The following chapter of twenty-six pages deals with transillumination of the cavities. The pathology and diagnosis of suppuration in the cavities each occupy a chapter; finally treatment is discussed in thirty-four pages.

The language throughout is clear, the illustrations copious and most admirably selected and executed, and the work as a whole will be found of great practical value. But while the earlier chapters are rather too full and detailed for students, and will appeal mainly to the rhinologist, the portions dealing with diagnosis and treatment are adapted for the student and practitioner, but are too elementary for the specialist. Thus there is a distinct want of proportion in the work, in our opinion the one and only serious fault to find with it.

